# Novel Applications of a Percutaneous Dual-Lumen Cannula Beyond a Right Ventricular Assist Device

**DOI:** 10.1155/crit/7507711

**Published:** 2025-09-15

**Authors:** Patrick Butler, Taylor Wilson, Jennifer Cloutier, Jean-François Légaré, Christopher W. White

**Affiliations:** ^1^Faculty of Medicine, Dalhousie Medicine New Brunswick, Saint John, Canada; ^2^Department of Anesthesia, New Brunswick Heart Center, Saint John, Canada; ^3^Division of Cardiac Surgery, New Brunswick Heart Center, Saint John, Canada; ^4^Department of Surgery, Dalhousie University, Halifax, Canada

## Abstract

The ProtekDuo's dual-lumen design combined with its ability to be inserted percutaneously allows for its use in a wide variety of clinical scenarios. Therefore, it was chosen to be used as the venous cannula on cardiopulmonary bypass to treat a patient who had a high risk of right ventricular failure and a need for mechanical circulatory support postoperatively. Biventricular failure occurred during the patient's course in hospital, and the ProtekDuo was used in a novel hybrid configuration using both the distal port on the ProtekDuo and the aortic cannula as outflow cannula for the extracorporeal membrane oxygenation circuit, and the proximal port on the ProtekDuo was used as the venous return. Gradually increasing ProtekDuo distal outflow and decreasing aortic cannula outflow facilitated an isolated and progressive loading of the left ventricle while maintaining support for the right ventricle. In total, four separate mechanical circulatory support configurations were used to support and optimize the patient's hemodynamics. The ability to utilize a single cannula in a hybrid manner may be useful in patients requiring controlled weaning of mechanical circulatory support.

## 1. Introduction

**Figure 1:**
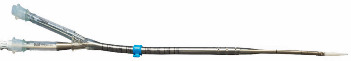
The ProtekDuo cannula.

Mechanical circulatory support (MCS) devices are being increasingly utilized in the treatment of cardiogenic shock. Device development has expanded the armamentarium available to treat advanced cardiorespiratory failure, including easily deployable percutaneously inserted options [1, 2]. The TandemLife ProtekDuo (Figure 1) is a percutaneously inserted dual-lumen cannula that can be adapted to provide cardiopulmonary support in a wide variety of clinical scenarios. We present a case where the ProtekDuo was used in four different configurations to support a single patient who required a pericardiectomy. Patient written consent for the publication of the study was not possible as the patient is deceased.

## 2. Case Presentation

A 56-year-old female was admitted to the hospital with anasarca. Her past medical history was significant for acute myelogenous leukemia (allogenic hematopoietic stem cell transplant), congestive heart failure secondary to an anthracycline reaction, chronic obstructive pulmonary disease, obesity, hypothyroidism, hypertension, and chronic kidney disease (creatinine clearance 43 mL/min). Investigations revealed a late diagnosis of constrictive pericarditis complicated by congestive hepatopathy and liver cirrhosis (Child–Pugh B).

**Figure 2:**
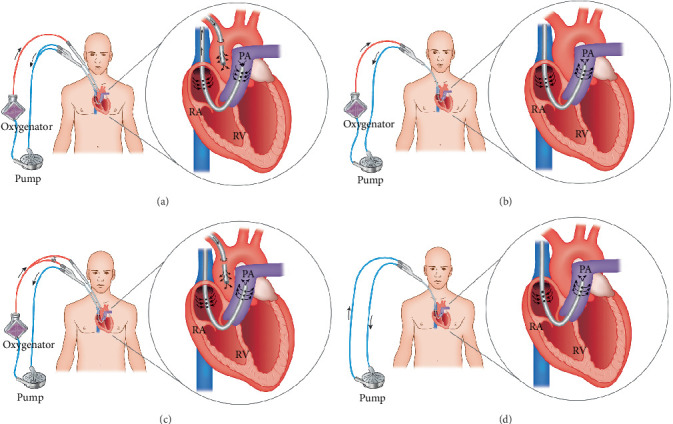
The ProtekDuo cannula utilized (a) as a drainage cannula for cardiopulmonary bypass and venoarterial extracorporeal membrane oxygenation (ECMO), (b) as a right ventricular assist device plus oxygenator, (c) in a hybrid configuration with venous drainage to the ECMO circuit via the proximal port of the ProtekDuo and outflow from the pump divided between the distal port of the ProtekDuo and the aortic cannula (a partial occlusion clamp is needed on distal port of the ProtekDuo cannula), and (d) as an isolated right ventricular assist device.

A pericardiectomy was scheduled. Due to the presence of multiple risk factors for right ventricular (RV) failure, a Swan–Ganz catheter was inserted via the left internal jugular vein preoperatively. The right neck was included in the sterile surgical field in preparation for potential ProtekDuo insertion. Under ultrasound guidance, a 7F sheath was placed in the right internal jugular vein, and a balloon wedge catheter was floated into the right main pulmonary artery (PA) under fluoroscopic guidance. Then, a sternotomy was performed, and the pericardiectomy was commenced; however, the patient developed RV failure necessitating cardiopulmonary bypass (CPB). An aortic cannula was placed in the ascending aorta. A double curved Lunderquist wire was advanced through the balloon wedge catheter and parked in the right PA. Following serial dilation (Opus Vascular Access Kit), a 29 French ProtekDuo cannula was advanced over the Lunderquist wire into the right PA. Both lumens of the cannula were deaired and connected to the venous line of the CPB circuit using a Y connector in a fashion similar to that used in a Dembitsky bridge (Figure 2a) [3]. The cannula was withdrawn gradually until the tip of the cannula transitioned into the main PA. Once the pericardiectomy was completed, the patient was weaned from CPB but developed RV failure. The ProtekDuo cannula was connected to an extracorporeal membrane oxygenation (ECMO) circuit, and 2.5 L per minute of right ventricular assist device (RVAD) support was initiated with drainage from RA and outflow into the PA (Figure 2b). This provided adequate left ventricular (LV) filling while maintaining a midline interatrial septum and a normal pulmonary capillary wedge pressure.

Despite initially doing well, during the first postoperative evening, the patient developed progressive hypotension, rising PA pressures, and increased lactate levels. An urgent transesophageal echocardiogram (TEE) demonstrated acute LV failure, a markedly dilated LV, and severe mitral valve regurgitation. She had evidence of pulmonary edema with frothy secretions appearing in the endotracheal tube and rising airway pressures. Cardiac arrest was imminent, and the sternum was reopened at the bedside. An arterial cannula was placed in the ascending aorta, and both lumens of the ProtekDuo were used as the venous cannula for venoarterial ECMO (Figure 2a). Central cannulation was chosen as reopening the chest was believed to provide more rapid arterial access. Furthermore, the severity of the pulmonary edema raised concerns regarding the possibility of North-South syndrome with peripheral cannulation, while simultaneously, the severity of the LV dysfunction on TEE suggested that a LV vent may have been required. By postoperative Day 5, there was evidence of LV recovery, and the ProtekDuo was employed in a hybrid fashion (Figure 2c). The proximal port was used for venous return to the ECMO circuit, and the ECMO outflow was delivered to the distal port (1 L per minute) and the aortic cannula (3 L per minute). This was controlled using a partial occlusion clamp on the distal port line. Over the next 3 days, flow through the distal port was increased, and flow through the aortic cannula was decreased. This hybrid approach facilitated progressive loading of the LV and weaning from venoarterial ECMO. On postoperative Day 9, an axillary intra-aortic balloon pump (IABP) was implanted, the aortic cannula was removed, and the patient was transitioned back to an RVAD plus oxygenator configuration (Figure 2b). By postoperative Day 13, the oxygenator was removed, and the patient was maintained on the RVAD (Figure 2d) and IABP. Daily physical rehabilitation eventually allowed the patient to mobilize. Unfortunately, while the RVAD and IABP were being weaned, the patient suddenly developed a distended abdomen, rising lactate, and rapidly progressive vasopressor requirements due to ischemic bowel. The patient did not wish to pursue abdominal surgical intervention, and she unfortunately expired.

## 3. Discussion

RV failure is a life-threatening postoperative complication that may develop following pericardiectomy. In extreme cases, MCS may be required as a bridge to recovery [4]. Use of the ProtekDuo as an RVAD and venous drainage cannula for ECMO has been described previously [2, 5, 6]; however, this report expands upon its additional applications by describing four distinct cannulation strategies on the same patient, without exchanging the original cannula. The dual-lumen design, combined with its upper body (right internal jugular vein) percutaneous insertion, facilitates early mobilization and physiotherapy postoperatively while the device is being weaned. Additionally, it may decrease the risk of infection compared to devices that require femoral insertion [2]. The ability to add and remove an oxygenator also increases the versatility of the cannula by providing pulmonary support, as was performed in this case. Its novel use in the hybrid configuration during centrally cannulated VA-ECMO (Figure 2c) may be helpful in patients with RV predominant biventricular failure where a gradual progression to isolated RVAD support is desired. Lastly, both lumens of the cannula can be utilized to facilitate excellent venous drainage during VA-ECMO or CPB.

## 4. Conclusion

This case exemplifies the versatility of a dual-lumen cannula and how the proximal and distal ports can be utilized in a variety of MCS configurations, which facilitate optimization of hemodynamics in a variety of different clinical scenarios (RV failure, biventricular failure, and pulmonary failure). The ability to use a single, percutaneous cannula in a hybrid manner may be useful in patients with RV predominant biventricular failure where a controlled progression to isolated RVAD support is desired.

## Data Availability

Data sharing is not applicable to this article as no datasets were generated or analyzed during the current study.

## Nomenclature

MCS: mechanical circulatory support
RV: right ventricle
CPB: cardiopulmonary bypass
ECMO: extracorporeal membrane oxygenation
RVAD: right ventricular assist device
LV: left ventricle
IABP: intra-aortic balloon pump
TEE: transesophageal echocardiogram
PA: pulmonary artery
RA: right atrium
